# Writing and reading with the longitudinal component of light using carbazole-containing azopolymer thin films

**DOI:** 10.1038/s41598-022-07440-9

**Published:** 2022-03-03

**Authors:** Alexey Porfirev, Svetlana Khonina, Nikolay Ivliev, Alexei Meshalkin, Elena Achimova, Andrew Forbes

**Affiliations:** 1 Image Processing Systems Institute-Branch of the Federal Scientific Research Centre Crystallography and Photonics of the Russian Academy of Sciences, Samara, 443001 Russia; 2 Samara National Research University, Samara, 443086 Russia; 3 Institute of Applied Physics, Chisinau, MD2028 Moldova; 4 School of Physics, University of the Witwatersrand, Johannesburg, 2050 South Africa

**Keywords:** Polymers, Laser material processing

## Abstract

It is well known that azobenzene-containing polymers (azopolymers) are sensitive to the polarization orientation of the illuminating radiation, with the resulting photoisomerization inducing material transfer at both the meso- and macroscale. As a result, azopolymers are efficient and versatile photonic materials, for example, they are used for the fabrication of linear diffraction gratings, including subwavelength gratings, microlens arrays, and spectral filters. Here we propose to use carbazole-containing azopolymer thin films to directly visualize the longitudinal component of the incident laser beam, a crucial task for the realization of 3D structured light yet remaining experimentally challenging. We demonstrate the approach on both scalar and vectorial states of structured light, including higher-order and hybrid cylindrical vector beams. In addition to detection, our results confirm that carbazole-containing azopolymers are a powerful tool material engineering with the longitudinal component of the electric field, particularly to fabricate microstructures with unusual morphologies that differentiate from the total intensity distribution of the writing laser beam.

## Introduction

The possibility of controlling the multiple degrees of freedom of light, including the amplitude-phase distribution and polarization state, has gained traction of late, for what is now referred to as structured light^1^. This in turn has opened up the way for many unique techniques and devices exploiting this new found control, including overcoming the diffraction limit in imaging with stimulated emission depletion (STED) microscopy^2–4^, optical trapping and tweezing to realize optically driven micro-rotors and micromechanical pump^5,6^, and enhanced materials processing with vectorial light^7,8^ to name just a few. Here, both two- dimensional (2D) and three-dimensional (3D) vectorially structured fields are exploited^9,10^, the former limited to transversally inhomogeneous light while the latter includes a longitudinal component to the electric field for 3D tailored light. While the creation of such 3D light fields is well established^11,12^, the detection of the longitudinal component of the electric field remains experimentally challenging, including nanotomographies^13–15^, scanning near-field optical microscopy (SNOM)^16–18^ and direct recording of the focal pattern in photoresist^19^. In the terahertz (THz) spectral region, it is possible to directly observe the longitudinal component and even obtain specific phase information for it using THz time-domain spectroscopy technique or electro-optic sampling^20,21^. However, in the visible and ultraviolet ranges, the short period of the electromagnetic waves significantly complicates this detection. This is in stark contrast to the ease with which one may visualize the transverse components of a light field - photo and video cameras record the intensity, different types of Shack-Hartmann sensors can detect the wavefront^22^, and polarimeters allow one to determine the local polarization direction in the transverse plane of the light field^23,24^. Rather than a special category of structured light, almost all optical beams exhibit the longitudinal component. In some cases, for example, under sharp focusing conditions, the larger part of light energy is exactly in the longitudinal component of the light field, where some of the aforementioned approaches fail altogether.

An emerging solution to this problem is the use of anisotropic materials that are sensitive to the polarization orientation of the illuminating radiation, for example, azobenzene-containing polymers (azopolymers)^25–29^. It is well known that the orientation of azobenzene molecules changes upon irradiation with polarized light, making them efficient and versatile materials for different photonic applications, such as the fabrication of linear diffraction gratings, including subwavelength gratings, microlens arrays, spectral filters, and many others^26,30^. This is a unique group of materials in which the photoisomerization reaction of azobenzene molecules induces the material transfer at both meso- and macroscale^31^. Under the illumination of polarized light, after light absorption, azobenzene molecules are redistributed perpendicular to the polarization of the light^27,28^. Although azo-polymer mass transfer induced by the longitudinal component of light has been demonstrated, the literature presents contradictory explanations for the mechanisms, while material choice has prohibited differentiation of the transverse and longitudinal components, making direct measurements of the longitudinal field strength problematic. Note that the observed effect of formation of the structure, the profile of which corresponds to the structure of the longitudinal component of the electric field can also occur for other photosensitive polymers. In this case, an important point is that the structures are not formed due to the longitudinal component of the electric field (it can be quite small). The longitudinal component is only convenient for approximating the structure/profile of the formed relief. Obviously, the longitudinal component of the electric field is completely determined by the complex distribution of the transverse components and the state of polarization. Therefore, in fact, it is the state of polarization (i. e. the ratio of the transverse components) that determines the shape of the formed structures, as noted in other works, including^32^.

Here we propose to use carbazole-containing azopolymer thin films to directly visualize the longitudinal component of the incident laser beam, a crucial task for the realization of 3D structured light yet remaining experimentally challenging. We demonstrate the approach on both scalar and vectorial states of structured light, including higher-order and hybrid cylindrical vector beams. In addition to detection, our results confirm that carbazole-containing azopolymers are a powerful tool material engineering with the longitudinal component of the electric field, particularly to fabricate microstructures with unusual morphologies that differentiate from the total intensity distribution of the writing laser beam. We show the new possibilities of using carbazole-containing azopolymer thin films to determine the characteristics of the polarization state in the case of both uniformly and non-uniformly polarized light fields (higher-order and hybrid cylindrical vector beams) as well as to encode information in the polarization state of the light. Moreover, we provide the qualitative model describing the profiles of the microstructures shaped under the illumination of a structured laser beam with the predetermined intensity and polarization distribution and discuss the possible mechanism of their formation.

**Figure 1:**
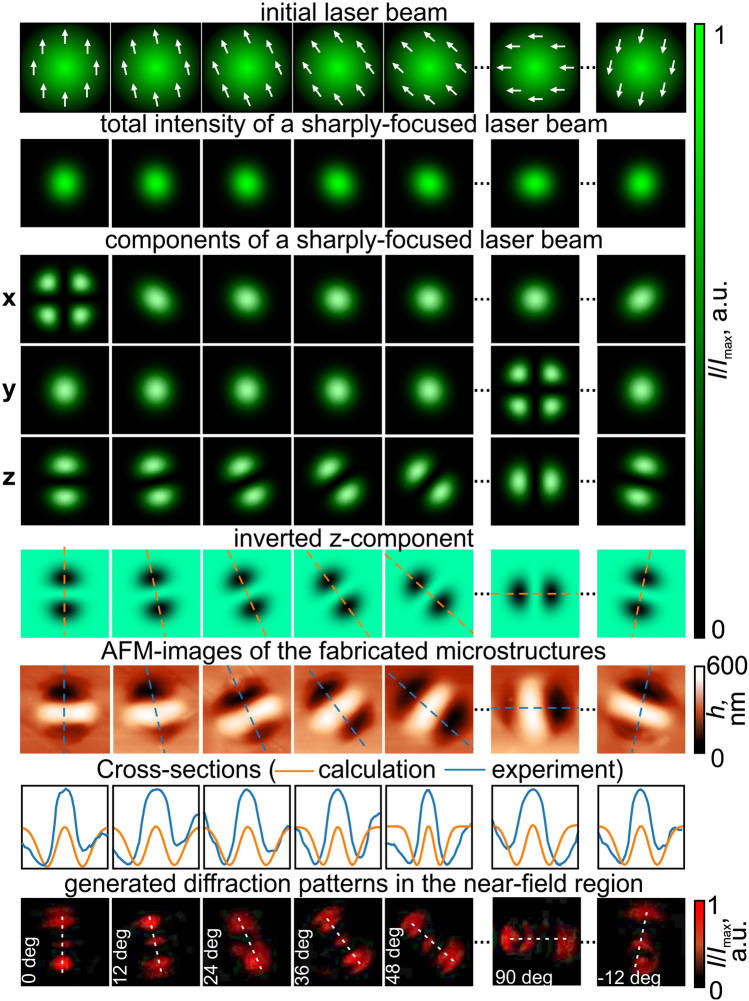
Linearly polarized laser beams for the structuring of carbazole-containing azopolymer thin films and exploitation of the fabricated microprotrusions that determine the polarization direction of the light. The white arrows in the initial laser beam intensity distributions show the linear polarization orientation. The white dashed lines in the generated diffraction patterns show the orientation of the line passing through the centers of the generated three light spots, coinciding with the polarization direction of the writing laser beam.

## Results

### Measurements of the parameters of uniformly-polarized laser beams

Figure 1 shows the total intensity distributions and individual electric field components of a sharply focused (NA = 0.85) linearly polarized Gaussian beam calculated using the Richards-Wolf equation^33^. A rotation of the polarization direction by an arbitrary angle leads to the rotation of all components by the same angle, resulting in the shaping of a rotated elongated light spot in the focal plane. Previously, it was presented that in the case of illumination of a trans-PMA-DR1 thin film using *x*-polarized laser beams, only *x*-component of the electric field contributes appreciably to the formation of the nanostructures^34^. Under the action of polarized laser radiation, the azobenzene molecules absorbing light are redistributed perpendicular to the polarization direction at the nanoscale while the material undergoes directional photoinduced motion at the microscale. The temporary molten azopolymer moved along the polarization direction from higher intensity to lower intensity.

However, our results obtained with carbazole-containing azopolymer thin films demonstrate the formation of the structures with other profiles. From the atomic force microscope (AFM) images presented in Fig. 1, the profiles of the shaped microstructures repeat the inverted structure of the *z*-component of the electric field. The irradiation intensity and the exposure time were 10 kW/cm$^2$ and 1 s, respectively, and the laser beam was focused on the film surface. During the experiments, the higher energy of incident laser radiation leads to the larger height of the formed central bump^35^. The similar relationship between the surface deformations and the longitudinal component of the electric field was mentioned by Grosjean and Courjon in the case of illuminating Bessel beams with radial, azimuthal, and circular polarization when PMMA-DR1 polymer used, which consists of azo-chromophore DR1^29^. The surface modulation was proportional to the longitudinal intensity; however, the concentrations of the material were mainly located in the maxima of the longitudinal component distribution, which contradicts all statements about the movement of the azopolymer from higher intensity to lower intensity. The results presented in Fig. 1 clearly demonstrate the movement of the carbazole-containing azopolymer from the areas of higher longitudinal component intensity to the areas of lower longitudinal component intensity. The difference between these results can be explained by the different structures of the azopolymer molecules used, as well as the different thicknesses of the films used. Either downward forces or upward forces dominate in a direction parallel to the polarization direction^36^. Thus, one can assume that the polarization sensitivity of carbazole-containing azopolymer molecules can initiate material transfer in accordance with the distribution of the intensity of *z*-component of light. Note, the contribution of the longitudinal component compared to the transverse components is insignificant: the ratio of the maximum intensity of the *z*-component to the maximum of the total intensity is 0.04.

Such polarization sensitivity of carbazole-containing azopolymers can be used for the implementation of polarization detectors, allowing the determination of the polarization direction. The last row in Fig. 1 demonstrates the diffraction patterns obtained when the illuminating linearly polarized Gaussian beam ($\lambda$ = 633 nm) is diffracted on the fabricated microstructures. The profiles of the fabricated microstructures are similar to the profiles of the degenerate, one-dimensional diffractive gratings or Hermite-Gaussian mode HG$_{02}$ formers^37,38^. The presented intensity distributions generated at the distance of approximately 10 μm from the thin film surface clearly demonstrate the formation of the corresponding three-light-spot patterns. From these distributions, anyone can determine the direction of the linear polarization from just one measurement. Moreover, this method is not sensitive to the fluctuations of the intensity of the incident laser radiation, since similar microstructures are formed in a wide range of used powers of the incident laser radiation that deforms the surface of the azopolymer thin film. Indeed, the formation of similar structures at the nanoscale was demonstrated with the laser radiation that had an intensity of 6.25$\sim$62.5 W/cm$^2$^34^. The presented results demonstrate the formation of a series of elongated microprotrusions rotated by 12 degrees relative to the adjacent structure. In this case, for a full 180-degree rotation, we can encode 15 different polarization states, each of which can encode a tetrad of one byte from 0001 to 1111. Then, the absence of the formation of the characteristic microprotrusion corresponds to 0000. As mentioned above, such microstructures were formed upon exposure to laser radiation with a power of 10 kW/cm$^2$ for 1 second; however, previously, it was shown that the type of surface deformation induced by the longitudinal component is weakly dependent on the exposure time^36^. Thus, we can expect the formation of similar microprotrusions with profiles repeating the inverted *z*-component of the electric field at noticeably shorter exposure times when using high-power laser systems, including pulsed laser systems.

**Figure 2:**
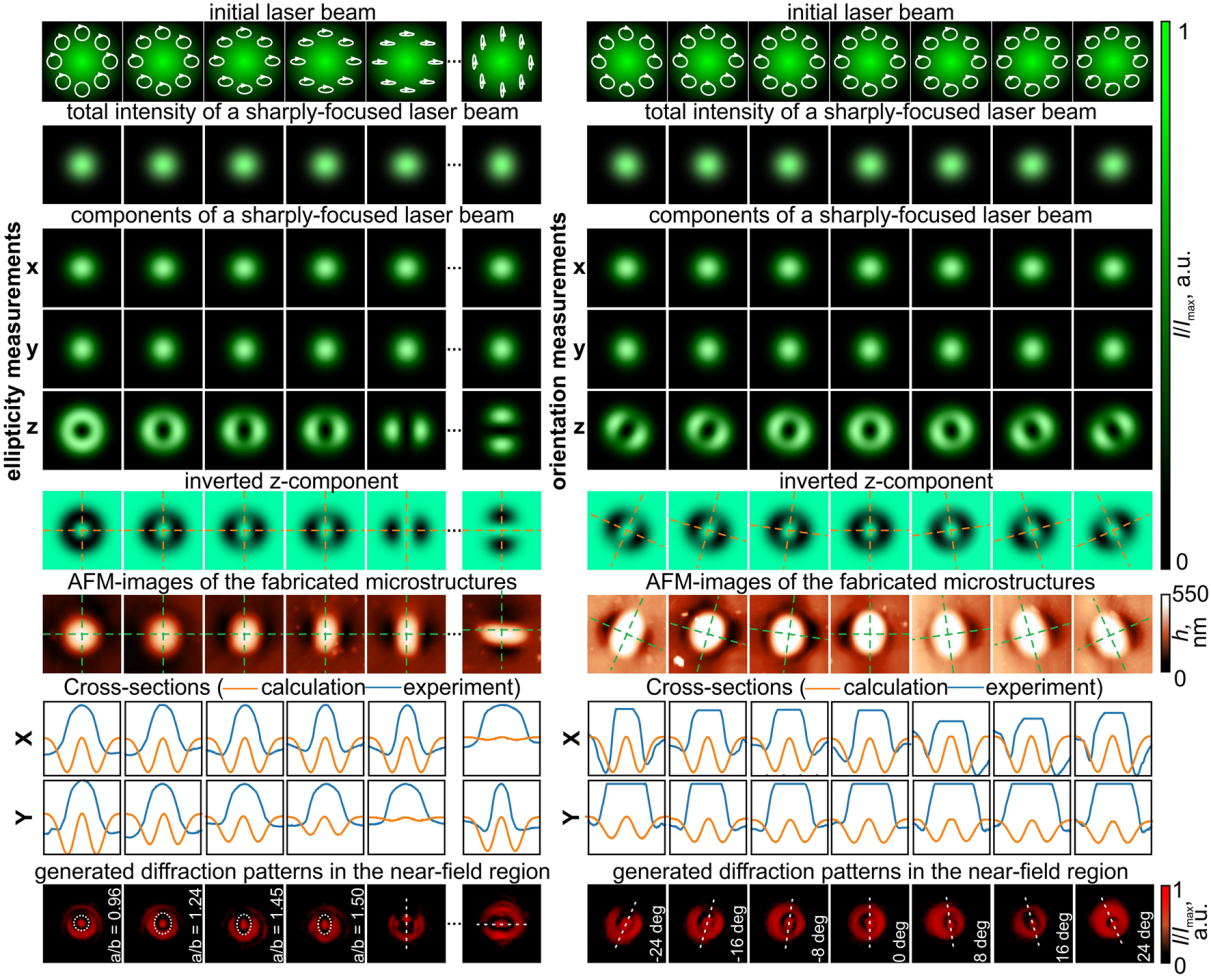
Elliptically polarized laser beams for the structuring of carbazole-containing azopolymer thin films and exploration of the fabricated microprotrusions that determine both the polarization direction of the light and the ellipticity of the polarization ellipse. The white circles in the initial laser beam intensity distributions represent the polarization ellipses. The white dashed ellipse in the generated diffraction patterns highlight the generated on-axis elliptical light spots and the white dashed lines show the orientation of the major axis of the generated elliptical light spots.

The use of other uniform polarization states, namely the circular/elliptical polarization, provides one additional degree of freedom for encoding information in comparison with the linear polarization. In this case, not only the orientation of the polarization ellipse but also the ellipticity can be used for information encoding. Figure 2 demonstrates these results.

The generated intensity distribution of the *z*-component of the electric field in the case of a circularly polarized Gaussian beam is a ring transforming into the contour of an ellipse (see a left group of images in Fig. 2). In this case, the ellipticity of the formed microprotrusions corresponds to the ellipticity of the polarization ellipse of the illuminating writing laser beam. In addition, the rotation of the polarization ellipse of the illuminating laser beam leads to the rotation of all three components of the electric field like in the case of the linearly polarized Gaussian beam. This rotation leads to the rotation of the formed elliptically-shaped microprotrusions on the surface of the carbazole-containing azopolymer thin film (see a right group of images in Fig. 2).

The experimentally generated diffraction patterns obtained as result of the diffraction of a linearly polarized Gaussian beam on the fabricated microstructures corresponds to the intensity distributions generated by elliptical apertures - the central elliptical spot with ellipticity corresponding to the ellipticity of the polarization ellipse of the writing laser beam. When the elliptical polarization of the writing laser beams degenerates to the linear polarization, the fabricated microprotrusions generate three-light-spot patterns. The orientation of the polarization ellipse of the writing laser beam can be easily determined from the intensity patterns of the laser beam diffracted on these structures (see the last row in the right group of images in Fig. 2). Then, it is possible to use the combination of these characteristics of elliptically polarized light, the polarization orientation, and ellipticity to encode more than one tetrad of a byte - for example, possibly determining at least four different ellipticities (like in the case shown in Fig. 2) allows one to encode two additional bits. The ellipticity of the microprotrusions and the generated diffraction patterns determine the two lowest (bit positions 0 and 1) bits in the number, while bits with positions from 2 to 5 can be encoded in the polarization direction (like in the case of linearly polarized writing Gaussian beam considered above). Previously the possibility to use ultrafast laser systems for processing azopolymers and one-shot writing of complex structures was shown^39^. The repetition rate of such systems is in the kHz to MHz range and we believe that this is sufficient for the implementation of optical encoding/decoding information systems.

**Figure 3:**
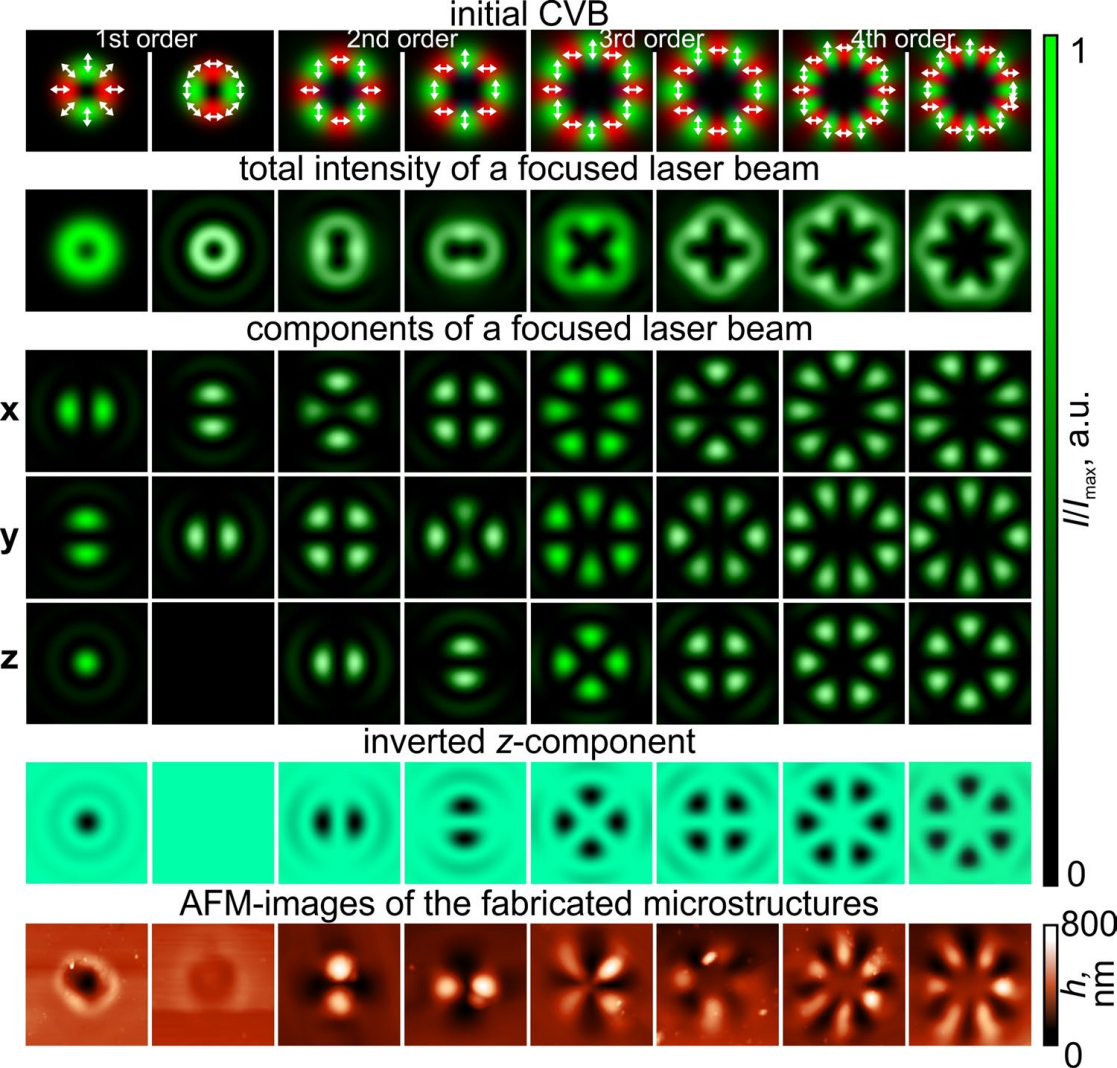
CVBs for the structuring of carbazole-containing azopolymer thin films. The white arrows in the initial laser beam intensity distributions represent the local orientation of the polarization vector.

### Detection of the polarization order of high-order cylindrical vector beams

The polarization sensitivity of carbazole-containing azopolymers allows one to not only determine the polarization characteristics of uniformly polarized laser radiation but also to determine the polarization order of non-uniformly polarized laser beams such as cylindrical vector beams (CVBs). As was mentioned above, the first order CVBs, azimuthally and radially polarized ones, were previously used for the formation of thin films of PMMA-DR1 polymer. However, the formation of only very simple structures such as nano- and micro dips and protrusions has been demonstrated^29,36^. It is well known that CVBs have an azimuthal symmetry not only in polarization structure but also in transverse intensity, which remains even in the case of focusing^40^. The total intensity of higher-order CVBs, taking into account the longitudinal component of the electric field, has a non-annular shape (see Fig. 3) that predicts the formation of complex nano- and microstructures, with structures representing *z*-component of a formed light field. Note, in this case, the contribution of *z*-component as compared to the transverse components increases as compared to the case of uniformly polarized beams: now the ratio of the maximum intensity of the longitudinal component to the maximum of the total intensity is 0.3 for radial polarization (a first-order CVB) and with an increase in the polarization order this value decreases up to 0.15 in case of fourth-order CVBs.The fabricated microstructures in the case of CVBs with orders from first to fourth are shown in the last row of Fig. 3. As was mentioned, the transverse intensity distributions of the focused CVBs have an annular shape while the fabricated microstructures are sets of alternating dips and protrusions located around the circumference. The number of dips and protrusions equals the number of extremums in the intensity distribution of the longitudinal component of the focused light field and equals to 4(*p*-1), p is the polarization order. That is less than the number of extremums in the intensity distributions of *x*- or *y*- components(which equals to 4*p*). The interesting situation is in the case of an azimuthally polarized laser beam (first-order CVB) with a zero longitudinal component of the electric field; a dip is formed, but the depth of this dip is about seven times smaller (60 nm versus 400 nm) than the depth of the dip formed in the case of the radially polarized laser beam. The profile of the formed structure replicates the structure of the total intensity distribution of a sharply focused laser beam - the material moves from the areas of high intensity on the generated light ring to the areas of low intensity in its center and along the outer border of the light ring.

The presented results for higher-order CVBs clearly demonstrate the possibility of determining the polarization order, as well as distinguishing the types of CVBs - in essence, whether the CVB used for laser writing is “radially” polarized(the inner polarization rotation of the beam 0 degrees) or “azimuthally” polarized(the inner polarization rotation of the beam 90 degrees). It is obvious that an arbitrary angle $\varphi _0$ (see Eq. (7) in Methods) can be chosen as the inner polarization rotation of the *p*th order CVB, and the orientation of the fabricated complex structures is defined by this angle $\varphi _0$. This is similar as the orientation of the microprotrusions formed using a linearly polarized Gaussian beam is defined by the polarization direction. Thus, it is possible to visualize and determine the polarization order and the inner polarization rotation angle of the used CVBs. The importance of possibly determining the polarization order is explained by the fact that CVBs have begun to be actively used in optical communication systems with mode division multiplexing. This allows the polarization order to be used as another degree of freedom for information encoding. In addition, it should be noted that the fabricated microstructures are in fact phase sinusoidal multi-sector plates whose binary versions are widely used for the generation of superpositions of vortex beams with opposite topological charges and orbital angular momentum (OAM) states in free-space communication systems^41,42^, as well as for the formation of hybrid higher-order cylindrical vector beams^43^. In fact, detecting the polarization order of CVBs is possible using film polarizers; however, they only allow one to see one of the transverse components of the intensity distributions. In addition, they do not allow the differentiation of positive polarization orders from negative ones, since the transverse components of the $\pm p$th CVBs are practically the same^43,44^. Note that the structure of the inverted *z*-component and the AFM topography images have some differences for the second-order CVB. This is due to the fact that the inverted *z*-component is only a simple and convenient approximation of the relief shape. For a more accurate model describing the relief formation, see the Discussion and conclusion section.

**Figure 4:**
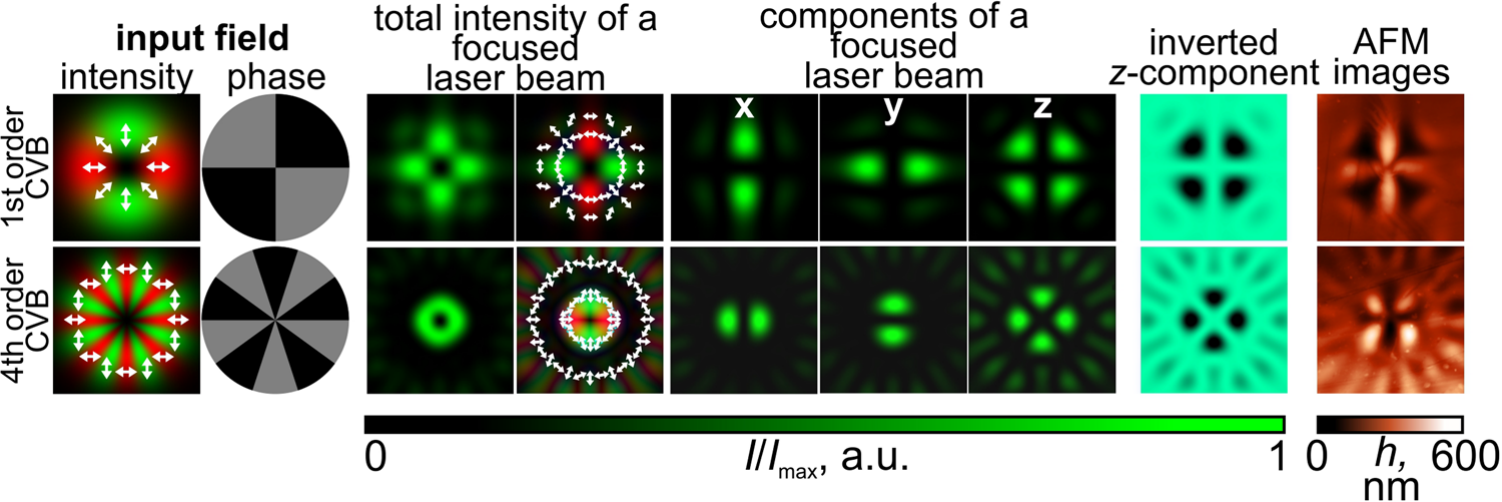
Hybrid CVBs for the formation of carbazole-containing azopolymer thin films. The white arrows in the initial laser beam intensity distributions and total intensity distribution of a focused laser beam represent the local orientation of the polarization vector.

### Detection of hybrid CVBs

Finally, the possibility of visualizing the longitudinal component of the hybrid higher-order CVBs was demonstrated. For the generation of such laser beams representing superpositions of CVBs with different orders and inner polarization rotation angles, combinations of CVBs with a fixed polarization order and a phase multi-sector plate with a $\pi$-phase shift between neighboring sectors are used^43^. The total intensity and polarization distributions of such laser beams have an azimuthal symmetry; however, the transverse intensity distributions have non-annular shapes. While *x*- and *y*- components of the electric field have different structures for different hybrid CVBs, the central part of the *z*-component is again a set of light maxima located around the circumference (see Fig. 4).

An interesting fact is that in these numerically and experimentally obtained results, the numerical aperture of the used microobjective focusing the generated hybrid CVBs on the surface of a carbazole-containing azopolymer thin film is only 0.5. This corresponds to the case of not sharp but rather moderate focusing conditions. In this case the energy of the longitudinal component is three times less than the energy of the transverse components of the field. However, the experimentally formed microstructures (especially in the case of focusing a fourth-order CVB passing through a ten-sector plate) clearly demonstrate the absolute matching of the formed microstructures’ profiles on the surface of the film and the distribution of the inverted longitudinal field component.

In the first case shown in the top row of Fig. 4, the structure of the hybrid CVB formed as a result of the focusing of an azimuthally polarized (a first-order CVB) laser beam passing through a phase four-sector plate is defined as a difference of a -1 order CVB, with the inner polarization rotation angle $\varphi _0$ equal to 90 degrees and a +3 order CVB with the same inner polarization rotation angle: ${\mathbf {e}}_{p=-1,\varphi _0=\pi /2} - {\mathbf {e}}_{p=3,\varphi _0=\pi /2}$^43^. In the second case shown in the bottom row of Fig. 4, a superposition of ${\mathbf {e}}_{p=-1,\varphi _0=0} - {\mathbf {e}}_{p=9,\varphi _0=\pi /2}$ was generated (with *p* = -1 in the central part and *p* = 9 in the peripherical area). Thus, it is possible to visualize the longitudinal component of the incident laser beam.

**Figure 5:**
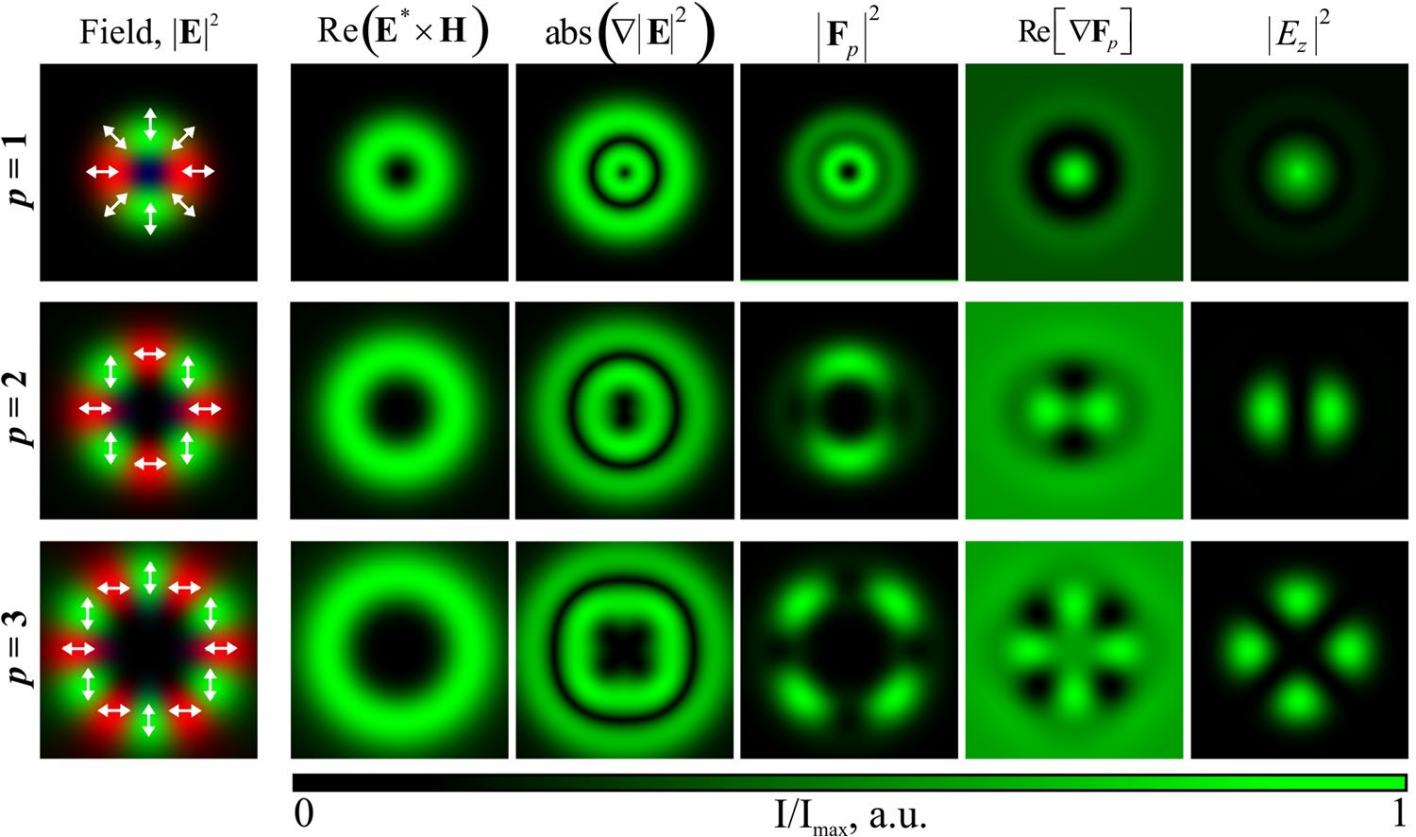
Comparison of the influence of the light field characteristics defined by Eqs. (1)–(4) on the structure of the formed relief in the case of the *p*th order illuminating CVBs.

## Discussion and conclusion

Unfortunately, today, there is no excellent theory that can completely describe the formation process of the nano- and microrelief on the surface of thin azopolymer films. However, in this work, we made an attempt to show the possibilities of visualizing the longitudinal field component using carbazole-containing azopolymer thin films and using them to determine the characteristics of the polarization state in the case of both uniformly and non-uniformly polarized light fields. The assumption put forward in this work about the key influence of the longitudinal field component on the profiles of microstructures formed on the surface of carbazole-containing azopolymer thin films is also confirmed by the results on the interference recording of one-dimensional diffractive gratings, shown earlier for interfering laser beams with different combinations of polarization states^45^. It has been repeatedly shown that the greatest contrast of the recorded gratings is observed when using two laser beams with orthogonal P:P polarizations, as opposed to beams with different combinations of polarizations (for example, two orthogonal S:S polarizations, or right- and left circular polarization). In all these cases, the longitudinal component has a similar structure, which matches the structure of the one-dimensional gratings formed on the surface of the film. Herewith, the longitudinal component of the field has the greatest energy just in the case of two laser beams with orthogonal P:P polarizations.

In addition, to explain the obtained results, we modeled several characteristics of the considered beams (see Fig. 5):

scattering force (proportional to Poynting vector):

1 $$\begin{aligned} {\mathbf {F}}_s\propto \text {Re} \left( {\mathbf {E}}^* \times {\mathbf {H}} \right) \end{aligned}$$

gradient force:

2 $$\begin{aligned} {\mathbf {F}}_g\propto \nabla |{\mathbf {E}}|^2 \end{aligned}$$

“polarization” non-gradient force^46^:

3 $$\begin{aligned} {\mathbf {F}}_p\propto \left( {\mathbf {E}}^* \cdot \nabla \right) {\mathbf {E}} \end{aligned}$$

divergence of polarization force defined by Eq. (3) is proportional to the structure of the formed relief, as shown in^47^:

4 $$\begin{aligned} h_p\propto \text {Re} \left[ \nabla {\mathbf {F}}_p \right] \end{aligned}$$

As can be seen from Fig. 5, the characteristic $\text {Re} \left[ \nabla {\mathbf {F}}_p \right]$ very well approximates the structure of the formed relief, while the structure of the longitudinal component $|E_z|^2$ is also close to the structure of the microrelief formed in the azopolymer thin film. Since it is much easier to calculate the distribution of $|E_z|^2$ than $\text {Re} \left[ \nabla {\mathbf {F}}_p\right]$, the intensity distribution of the longitudinal component can be used as the expected profile of the formed structure.

It should be noted that in Ref.^48^, a model was proposed for describing the formed microrelief based on the following expression:

5 $$\begin{aligned} h_m(x, y){\propto }(c_1 + c_2) M_0 + c_1 M_1 + c_2 M_2 + c_3 M_3 + c_B M_B \end{aligned}$$

where $$M_0 = \frac{\partial ^2}{\partial x^2} |E_x|^2 + \frac{\partial ^2}{\partial y^2} |E_y|^2,$$ $$M_1 = \frac{\partial ^2}{\partial x^2} |E_y|^2 + \frac{\partial ^2}{\partial y^2} |E_x|^2,$$ $$M_2 = \frac{\partial }{\partial x} \frac{\partial }{\partial y} (E_y^* E_x + E_x^* E_y),$$ $$M_3 = \nabla ^ 2 |E_z|^2 = \frac{\partial ^2}{\partial x^2} |E_z|^2 + \frac{\partial ^2}{\partial y^2} |E_z|^2,$$ $$M_B = \frac{\partial }{\partial x} \text {Re}(E_z^* E_x) + \frac{\partial }{\partial y} \text {Re}(E_z^* E_y).$$

**Figure 6:**
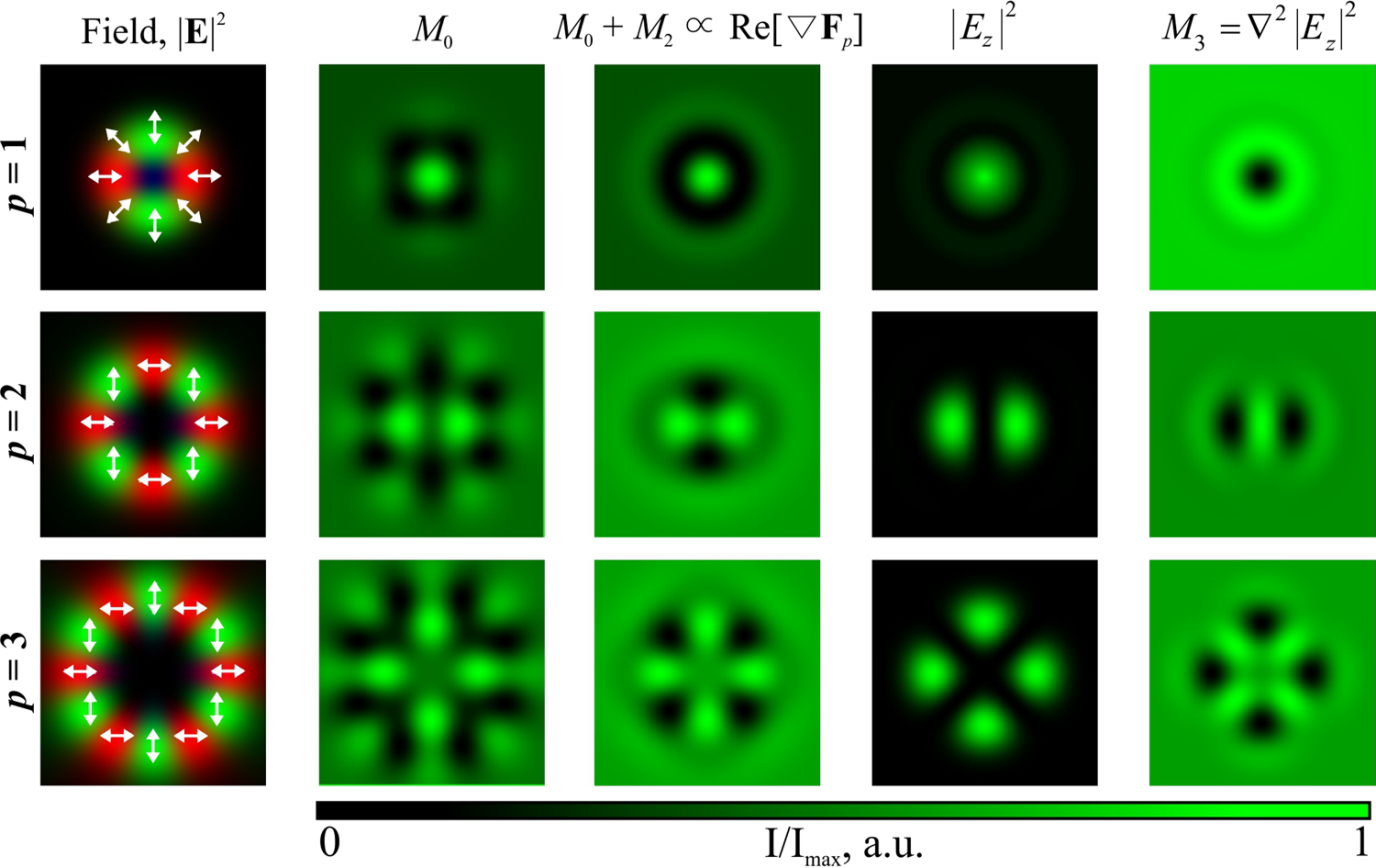
Comparison of the influence of various components of the model defined by Eq. (5) on the formation of the relief in the case of the *p*th order illuminating CVBs.

A comparative study of the influence of various components of the model defined by Eq. (5) on the structure of the experimentally formed relief showed that the most significant term is $M_3 = \nabla ^ 2 |E_z|^2$ , which actually corresponds to the inversion of the intensity of the longitudinal component, as well as the sum of two terms $M_0 + M_2 {\propto } \text {Re} \left[ \nabla \mathbf{F }_p\right]$ , which is close to the value defined by Eq. (4). As can be seen from Fig. 6, the structure of the inverted longitudinal component is very close to the structure of the model defined by Eq. (4) (up to inversion). Therefore, taking into account the simplicity of the calculation the inverted longitudinal component can be used as an approximate model of the formed relief.

These considerations can also explain the weak dependence of the spiral-shaped microstructures formed on an azobenzene-containing polymer film illuminated by vortex beams with different topological charges that Ambrosio et al. had demonstrated in 2012^46^. Analysis of the distributions of the longitudinal field component outside the focal area shows it matches with the profiles of the spiral-shaped microstructures formed in the case of linearly polarized optical vortices and predicts the impossibility of spiral mass transport in the case of circularly polarized optical vortices.

The examples presented by us demonstrate that carbazole-containing azopolymers are in fact a powerful tool for exploiting the longitudinal component of the electric field when it is necessary to fabricate microstructures with unusual morphologies that are different from the total intensity distribution of the writing laser beam. In particular, we offer an approach for writing with and detecting the longitudinal field component in vectorial light distributions, which we hope will inspire further research into the exciting interface of structured light and structured matter.

**Figure 7:**
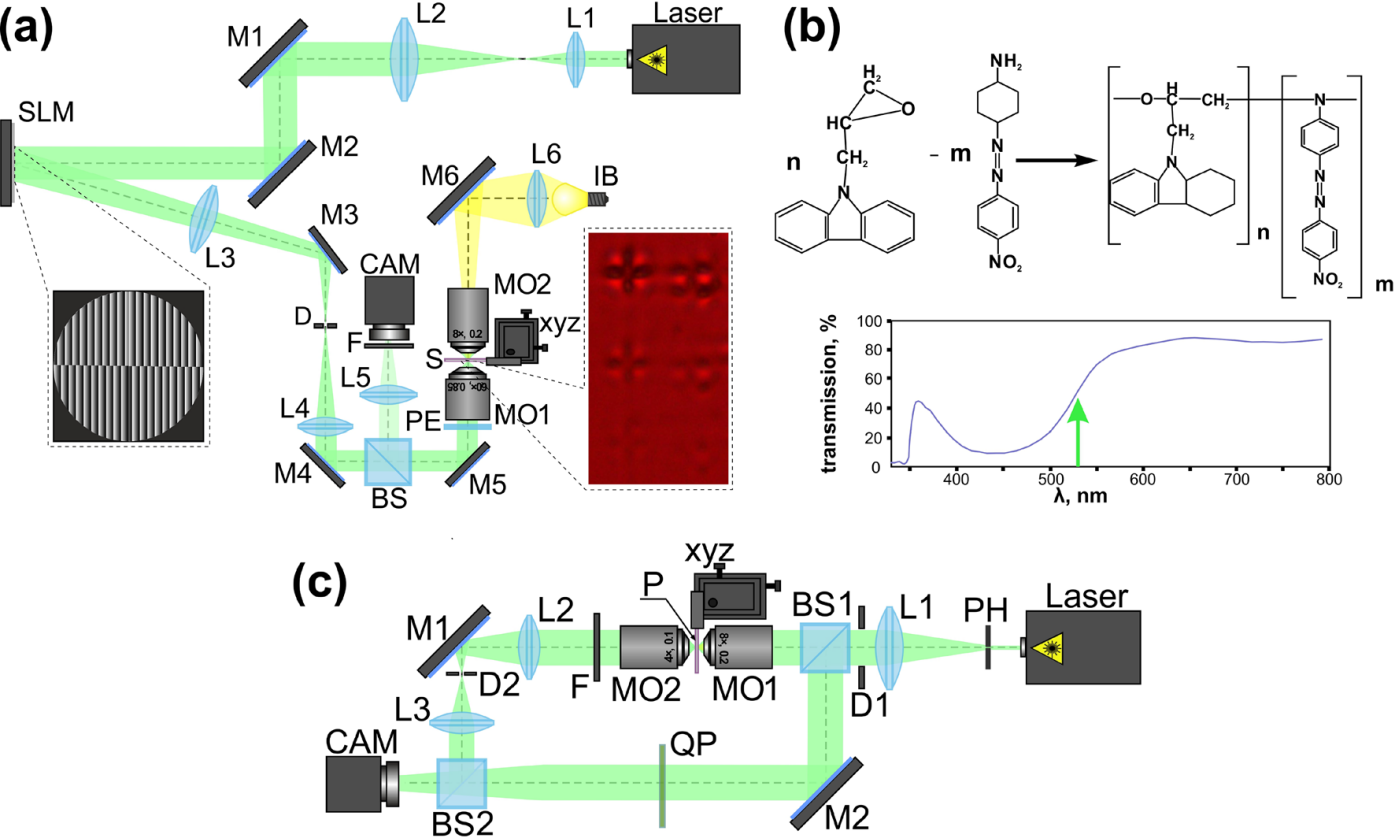
Laser writing and analyzing of microstructures on the surface of the carbazole-containing azopolymer thin film. (**a**) Optical setup for laser writing of microstructures on the surface of the carbazole-containing azopolymer thin film. Insets show an example of a phase mask of a multi-sector plate used in the experiments for the transformation of a CVB into a hybrid CVB and an optical microscopy image of the microstructures fabricated on the surface of the carbazole-containing azopolymer thin film using a second-order CVB. (**b**) Scheme of synthesis and chemical structure of EPC:DO3 and transmission spectra of EPC:DO3 azopolymer film. Green arrow indicates wavelength of recording laser (532 nm). (**c**) Optical setup for optical characterization of the fabricated microstructures on the surface of the carbazole-containing azopolymer thin film.

## Methods

### Direct laser writing setup

For the realization of direct laser writing of microstructures on the surface of carbazole-containing azopolymer thin films, we used an optical setup based on a reflective spatial light modulator (SLM) HOLOEYE PLUTO VIS (1920$\times$1080 pixels, pixel size of 8 μm) which is shown in Fig. 7a. A linearly polarized Gaussian beam from a solid-state laser ($\lambda$ = 532 nm) was extended and collimated with a combination of two lenses L1 and L2 with the focal lengths of 25 and 150 mm. The output laser power was varied in the range from 15 to 35 mW depending on the structure of the laser beam used for laser writing of microstructures. The collimated laser beam was directed onto the SLM with the help of mirrors M1 and M2, and the modulated reflected laser beam was spatially filtered with a combination of two lenses L3 and L4 with focal lengths of 500 and 150 mm, as well as a circular diaphragm D. The mirrors M3, M4, and M5, were used to direct the modulated laser beam into the input pupil of a micro-objective MO1 (NA = 0.85 for the experiments with uniformly polarized Gaussian beams and NA = 0.5 for the experiments with higher-order CVBs and hybrid CVBs). The glass substrate S with a carbazole-containing azopolymer thin film mounted on the 3-axis XYZ translation stage was located in the focal plane of the micro-objective MO1. A system consisting of a light bulb IB, a spherical lens L6 (focal length of 50 mm), a mirror M6, and a micro-objective MO2 (NA = 0.1) was used to illuminate the surface of the glass substrate. A polarization transformer (a half-wave plate, a quarter-wave plate, or a first/second-order *q*-plate depending on the experiment) was located before the micro-objective MO1 to transform initial *x*-polarized laser radiation into a linear polarization with the desired orientation, circular/elliptical polarization, or a cylindrical polarization of the first and second orders. For the generation of higher-order CVBs, combinations of *q*-plates and half-wave plates were used^49^. A beam splitter BS, lens L5 (focal length of 150 mm), and a video camera CAM (TOUPCAM UHCCD00800KPA; 1600$\times$1200 pixels, with a pixel size of 3.34 μm) were used to observe the surface of the glass substrate with the carbazole-containing azopolymer thin film during laser writing. A neutral density filter F was used to decrease the intensity of the observed laser beam.

The SLM was utilized for the realization of phase masks of multi-sector plates which are used for the generation of hybrid CVBs. In the experiments with uniformly-polarized (linearly, circularly, or elliptically-polarized) Gaussian laser beams or with higher-order CVBs, the SLM was applied only for the realization of blazed diffractive gratings guiding the shaped laser beam in the first diffraction order.

### Preparation of azopolymer thin films

Carbazole-based polymer 9-(2,3-epoxypropyl)carbazole (EPC) and azodye Disperse Orange 3 (DO3) were utilized in this study. DO3 was purchased as a commercial product from Sigma-Aldrich Company^50^. DO3 molecules were chemically attached to EPC oligomer by polycondensation scheme at a temperature of 120$^\circ$ C for 4 hours. The molar ratio between EPC and DO3 was 90/10. The synthesized copolymer EPC:DO3 was purified by precipitation in hexane and then in methanol. Scheme of synthesis and chemical structure of EPC:DO3 is presented in Fig. 7b.

The thin polymer films were prepared from the homogeneous copolymer solution by spin coating procedure using programmable spin-coater “SGS Spincoat G3P-8”^51^. The thickness of the polymer film was varied by changing the concentration of polymer solution and the rotation speed of spin coating. In this work, polymer thin films were prepared by spin-coating of the 100 wt. % polymer solutions in toluene onto a glass substrate. Orientation conditions for polymer solution deposited in 5 cm diameter optical glass substrate (BK7) were as follows: 1 cm$^2$ of liquid dispensed on the disk at rest, subsequently accelerated in about 3 s to 800 rpm, and spun for 60 s. Obtained films were dried in an oven at 60$^\circ$ C for 6 hours. The film thickness was determined by a microinterferometer microscope MII-4 with developed software^52^ and was around 1.20 μm. Figure 7b shows the UV-Vis transmission spectra for resulting azopolymer film EPC:DO3. The absorption maximum consists of a wide band peak centered in 450 nm with a FWHM of 120 nm. At 532 nm (the recording laser wavelength) azopolymer film has transmission about 50%, that allows the entire volume of the film to be used.

Thin films obtained by spin-coating method were checked by experimental apparatus for investigating photo-induced anisotropy using pump-beam-induced light at 473 nm and probe-wavelength wavelength at 650 nm. It was demonstrated that that thin azopolymer films before pump light irradiation are optically isotropic in red light.

### Profile measurements and analysis

For the measurements and analysis of the structure of the fabricated microstructures, we used a scanning probe microscope NT-MDT SOLVER Pro-M. In this case, a semi-contact operating mode was used, which is characterized by a minimal mechanical effect of the silicon probe on the sample surface. The tip curvature radius of the probe did not exceed 10 nm. The line scanning frequency was 0.5 Hz, with a study area of 20$\times$20 μm. The speed of movement of the silicon probe corresponding to the indicated parameters, at a resonant frequency of its oscillations of 235 kHz, made it possible to measure the profile of microstructures without geometric distortion in a reasonable time.

### Optical characterization

Figure 7c shows the optical setup used in the experiments for the optical characterization of the fabricated microstructures and analysis of the light fields generated by them. A linearly polarized Gaussian laser beam ($\lambda$ = 633 nm, P$_{out}$ = 1 mW) collimated using a pinhole PH (aperture size 40 μm) and a lens L1 (focal length 350 mm) was focused by a microobjective MO1 (3.7$\times$, NA = 0.1) onto the surface of the glass substrate with fabricated microstructures in a carbazole-containing azopolymer thin film (P). A diaphragm D1 was used both to separate the central spot of the Airy disk resulting from the wave diffraction of the pinhole and to adjust the laser beam diameter. Using a 3-axis XYZ translation stage, we moved the glass substrate and analyzed different microstructures. The intensity distributions of the generated beams were imaged using a microobjective MO2 (16$\times$, NA = 0.35), lenses L2 and L3 with focal lengths of 150 and 35 mm, and a video camera CAM (TOUPCAM UHCCD00800KPA; 1600$\times$1200 pixels, with a pixel size of 3.34 μm) to capture the images.

### Numerical simulation

Numerical calculations of total intensity distributions in the focal plane and distributions of different components of the electric field of the investigated focused laser beams were performed using the Richards-Wolf equation^33^. In this case, the electric field components of a monochromatic electromagnetic wave can be calculated as follows:

6 $$  \begin{aligned}   E(\rho ,\varphi ,z) =  & \left( {\begin{array}{*{20}c}    {E_{x} (\rho ,\varphi ,z)}  \\    {E_{y} (\rho ,\varphi ,z)}  \\    {E_{z} (\rho ,\varphi ,z)}  \\   \end{array} } \right) =  - \frac{{if}}{\lambda }\int_{0}^{\alpha } {\int_{0}^{{2\pi }} {\left( {\begin{array}{*{20}c}    {\left[ {1 + \cos ^{2} \phi (\cos \theta  - 1)} \right]} & {\sin \phi \cos \phi (\cos \theta  - 1)}  \\    {\sin \phi \cos \phi (\cos \theta  - 1)} & {\left[ {1 + \sin ^{2} \phi (\cos \theta  - 1)} \right]}  \\    { - \sin \theta \cos \phi } & { - \sin \theta \sin \phi }  \\   \end{array} } \right)} } \left( {\begin{array}{*{20}c}    {c_{x} (\phi )}  \\    {c_{y} (\phi )}  \\   \end{array} } \right) \\     &  \times B(\theta ,\phi )T(\theta )\exp [ik(\rho \sin \theta \cos (\phi  - \varphi ) + z\cos \theta )]\sin \theta d\theta d\phi , \\  \end{aligned}    $$

where $(\rho , \varphi , z)$ are the cylindrical coordinates in the focal region, $(\theta , \varphi )$ are the spherical angular coordinates of the focusing system’s output pupil, $\alpha$ is the maximum value of the azimuthal angle related to the system’s numerical aperture, $B(\theta ,\varphi )$ is the transmission function, $T(\theta ) = \sqrt{\cos \theta }$ is the pupil’s apodization function of aplanatic systems, $k = 2\pi /\lambda$ is the wavenumber, $\lambda$ is the wavelength, *f* is the focal length, $C(\varphi ) = (c_x(\varphi ), c_y(\varphi ))^T$ is the polarization vector of the initial light field.

A polarization vector of linearly and circularly/elliptically polarized Gaussian beams are well known^53^, and in the case of a *p*th order CVB with the inner polarization rotation angle of $\varphi _0$, the polarization vector has the following form:

7 $$\begin{aligned} {\mathbf {C}}(\phi )=\begin{pmatrix} \cos (p \phi + \phi _0)\\ \sin (p \phi + \phi _0)\\ \end{pmatrix}. \end{aligned}$$
